# Supplementary material for: Combination of in vivo proximity labeling and co-immunoprecipitation identifies the host target network of a tumor-inducing effector in the fungal maize pathogen Ustilago maydis
Source: J Exp Bot. 2023 May 24;74(15):4736–50. doi: 10.1093/jxb/erad188 (PMC10433927; doi:10.1093/jxb/erad188)
Supplement: erad188_suppl_Supplementary_Figures_S1-S7 [file erad188_suppl_supplementary_figures_s1-s7.pdf]

Combination of *in-vivo* proximity labeling and co-immunoprecipitation identifies the host target network of a tumor-inducing effector in the fungal maize pathogen *Ustilago maydis*

Wei Shi [1,4], Sara C Stolze [2], Hirofumi Nakagami [2,3], Johana C. Misas Villamil [1,4], Isabel ML Saur [1,4], and Gunther Doehlemann [1,4]

1. Institute for Plant Sciences University of Cologne, Cologne, D-50674, Germany
2. Protein Mass Spectrometry, Max-Planck Institute for Plant Breeding Research, Carl-von-Linné-Weg 10, 50829 Cologne, Germany
3. Basic Immune System of Plants, Max-Planck Institute for Plant Breeding Research, Carl-von-Linné-Weg 10, 50829 Cologne, Germany
4. Cluster of Excellence on Plant Sciences (CEPLAS)

Author for correspondence: Gunther Doehlemann (email: g.doehlemann@uni-koeln.de); ORCID: 0000-0002-7353-8456.

Supporting Figures S1 – S7

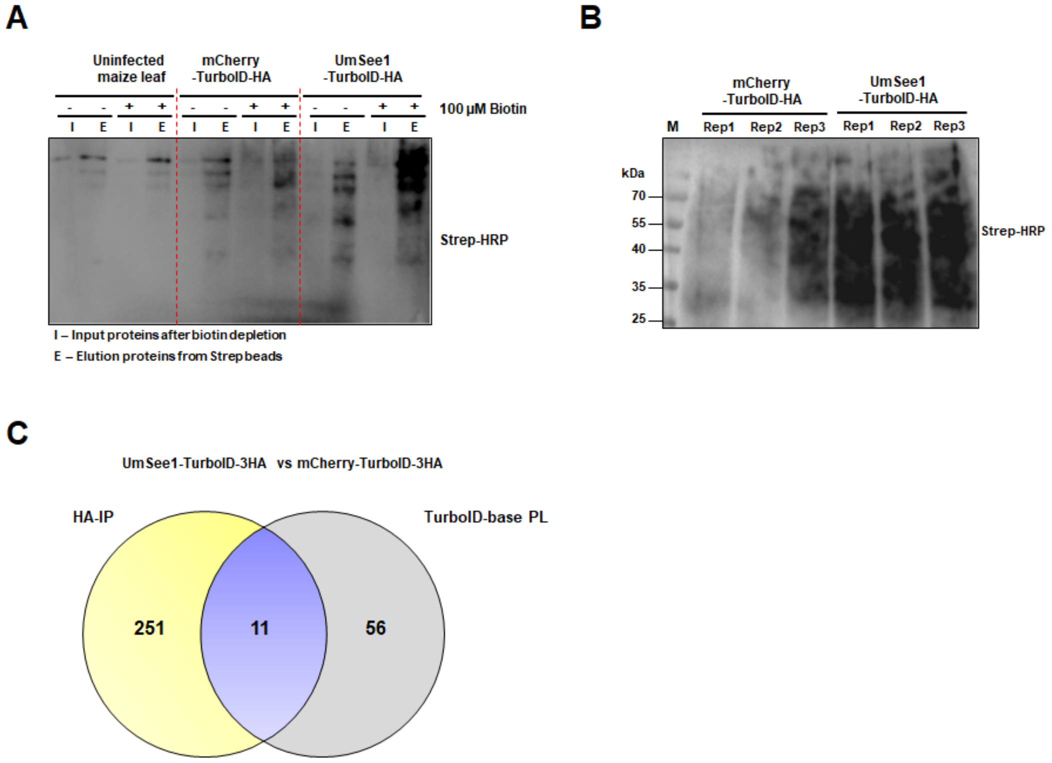

Fig S1.: UmSee1 interacting proteins enrichment by TurboID-based biotin labelling. (A) Analysis of biotinylated proteins in infected and non-infected leaves. Seven-day-old maize seedlings were infected with *U. maydis* mCherry-TurboID-HA/SG200 or UmSee1-TurboID-HA/ΔSee1. Non-infected leaves were used as a negative control. Two days post-infection non-infected and infected leaves were treated with (+) or without (-) 100 µM biotin. Infected leaves were harvested one-day post biotin treatment for protein extraction and Streptavidin-based precipitation of biotinylated proteins. (**B**) TurboID pull-down of infected *U. maydis* leaves. Biotinylated proteins of three biological replicates (Rep 1-3) were detected using Strep-HRP and further subjected for LC-MS/MS analysis. (**C**) Venn diagrams depicting the proteins that were specifically identified in UmSee1 samples by HA pull-down and TurboID-based biotin labelling.

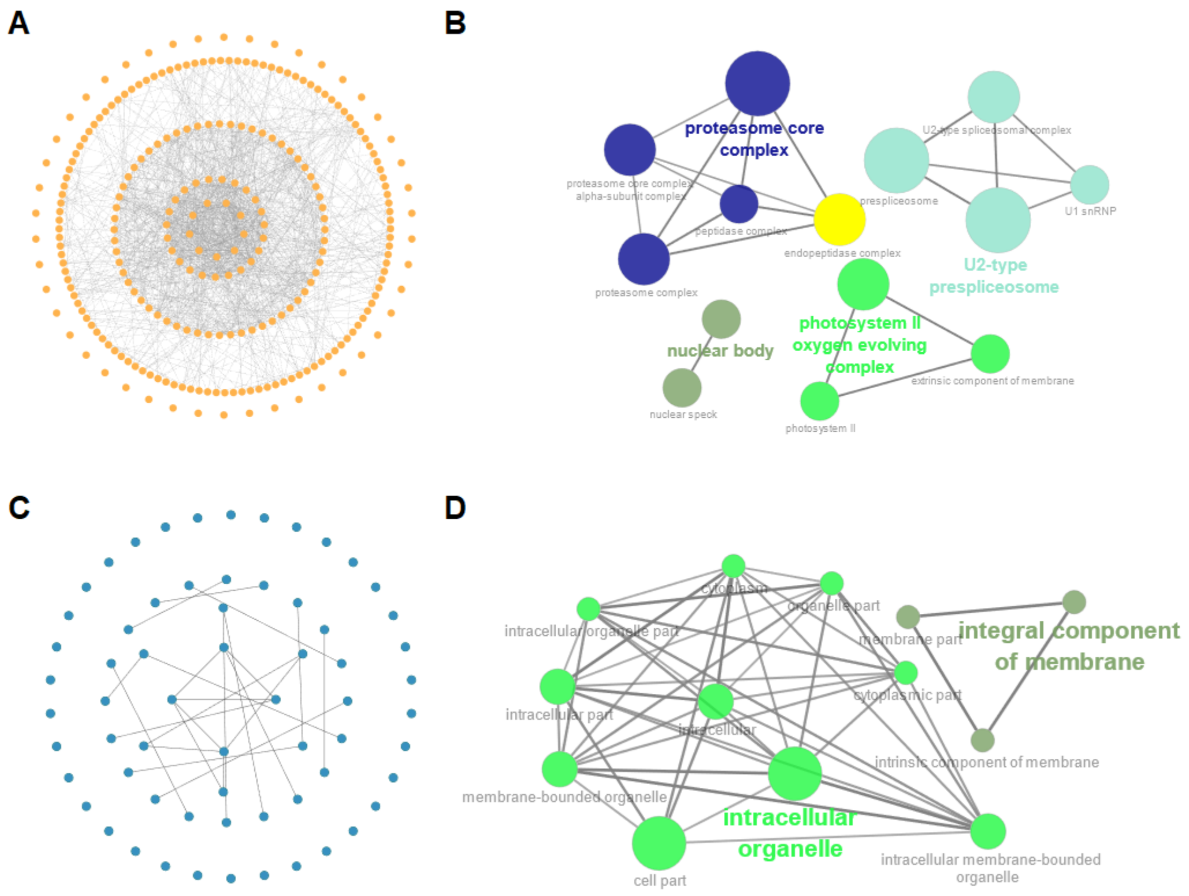

Fig S2.: Protein-protein interactions (PPIs) analysis of proteins that were specifically identified in UmSee1 samples. (**A**) Protein-Protein interaction (PPI) analysis of the identified UmSee1 interacting proteins using the STRING database. Significantly enriched UmSee1 interactors from HA-IP were submitted to the STRING database, and the protein interaction network was modified and analyzed by Cytoscape software (version 3.9.1). Edges represent protein-protein associations, including known interactions and predicted interactions (gene neighbourhood, gene fusions, gene co-occurrence). Proteins are represented by their gene ID. (**B**) Cluster analysis of the Protein-Protein interactions network of UmSee1 interactors from the HA-IP dataset by CluePedia (version 1.5.9) and ClueGo (version 2.5.9). (**C**) Protein-Protein interaction (PPI) analysis of the identified UmSee1 interacting proteins using the STRING database. Significantly enriched UmSee1 interactors from TurboID-based PL-IP were submitted to the STRING database, and the protein interaction network was modified and analyzed by Cytoscape software (version 3.9.1). Edges represent protein-protein associations, including known interactions and predicted interactions (gene neighbourhood, gene fusions, gene co-occurrence). Proteins are represented by their gene ID. (**D**) CluePedia (version 1.5.9) and ClueGo (version 2.5.9)- based cluster analysis of the Protein-Protein interactions network of proteins that were specifically identified in UmSee1 samples in the TurboID-based PL dataset.

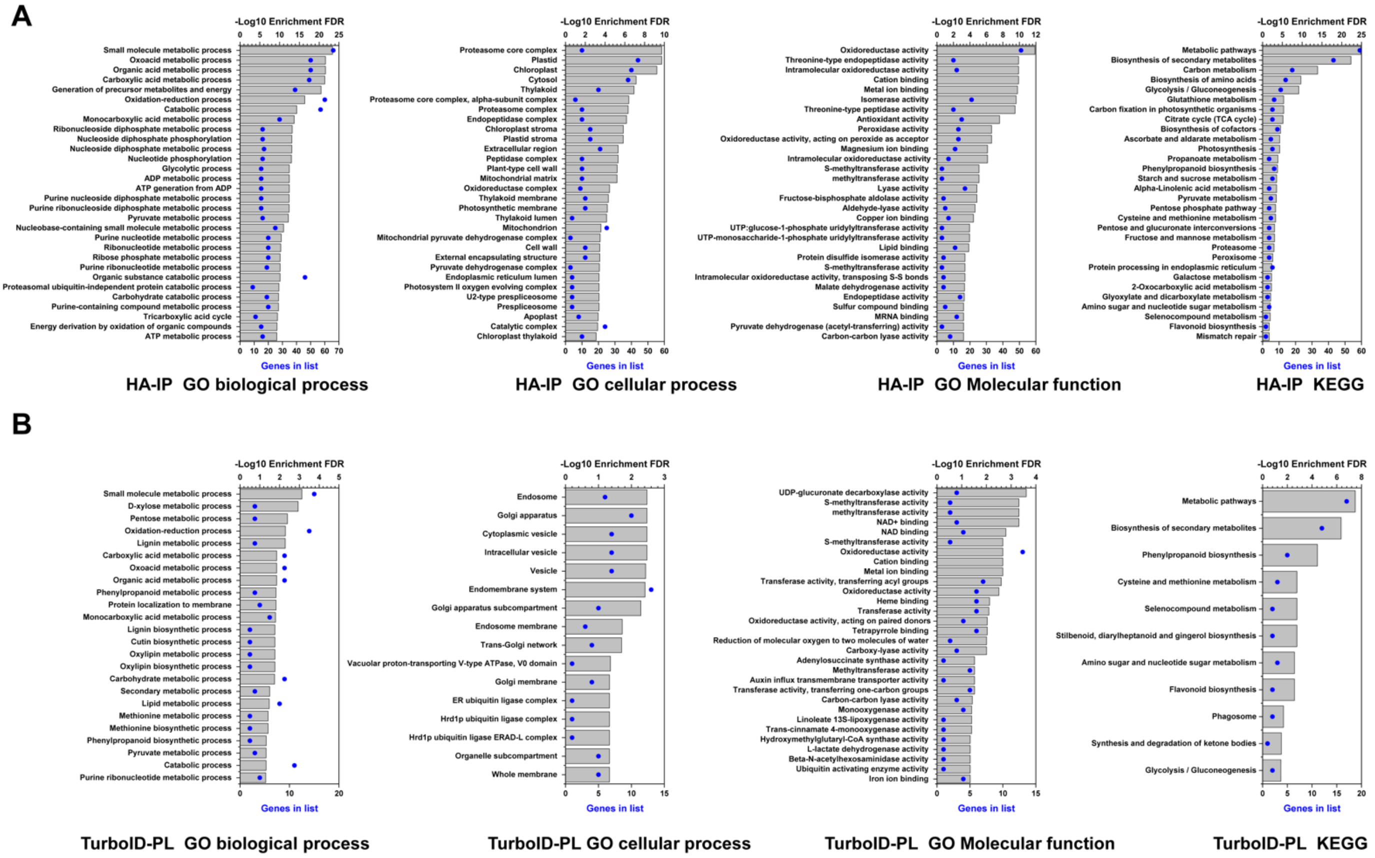

Fig S3.: Gene Ontology (GO) analysis of proteins that were specifically identified in UmSee1 samples. (**A**) Gene Ontology (GO) biological, cellular component, molecular function and KEGG enrichment of the identified UmSee1-interacting proteins from the HA-IP dataset using ShinyGO v0.66. (**B**) ShinyGO v0.66-based gene ontology (GO) of biological, cellular component, molecular function and KEGG enrichment of the proteins that were specifically identified in UmSee1 samples in the TurboID-based PL dataset.

A

Name	Protein ID	Funtion
ZmSIP1	Zm00001eb157120	Ubiquitin-conjugating enzyme E2 36
ZmSIP2	Zm00001eb369210	Proteasome subunit alpha type 3
ZmSIP3	Zm00001eb185960	AAA-type CDC48 protein

B

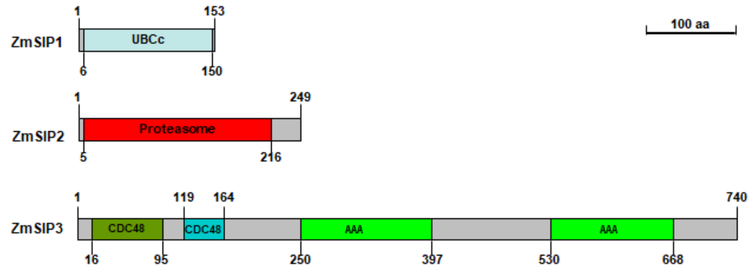

C

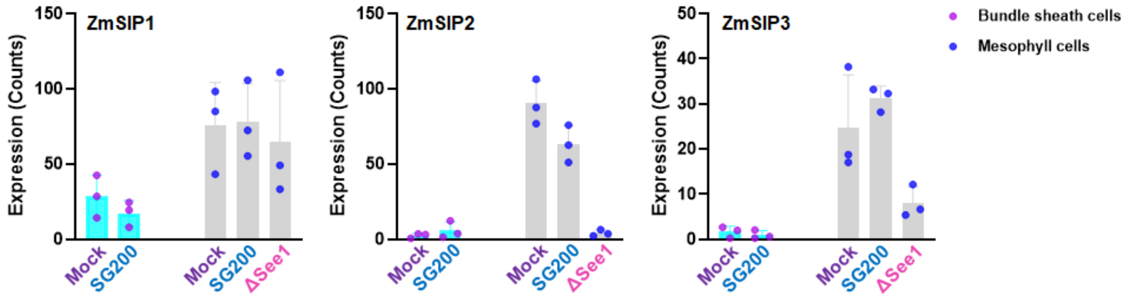

Fig S4.: Identified ZmSIPs (ZmSIP1, ZmSIP2, ZmSIP3). (**A**) ZmSIPs protein ID and putative function (**B**) Diagrams illustrating the protein domain structure of ZmSIP1, ZmSIP2 and, ZmSIP3. (**C**) Expression patterns of the ZmSIP1, ZmSIP2, and ZmSIP3 in different cell type of *U. maydis*-infected maize. Data extracted from the previously published RNA-seq (Villajuana-Bonequi *et al.*, 2019b).

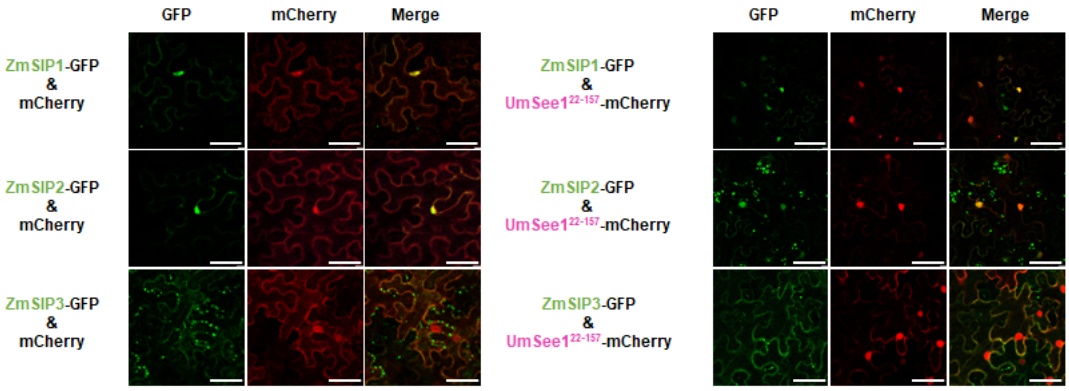

Fig S5.: Subcellular localization of ZmSIPs and UmSee1 upon expression in *Nicotiana benthamiana*. UmSee1ΔSP-mCherry, mCherry and ZmSIPs (ZmSIP1, ZmSIP2, ZmSIP3)-GFP were transiently co-expressed in *N. benthamiana* leaves. At three days post-Agrobacterium-mediated transformation fluorescence signals were observed by a Leica TCS SP8 confocal laser scanning microscope (Leica).

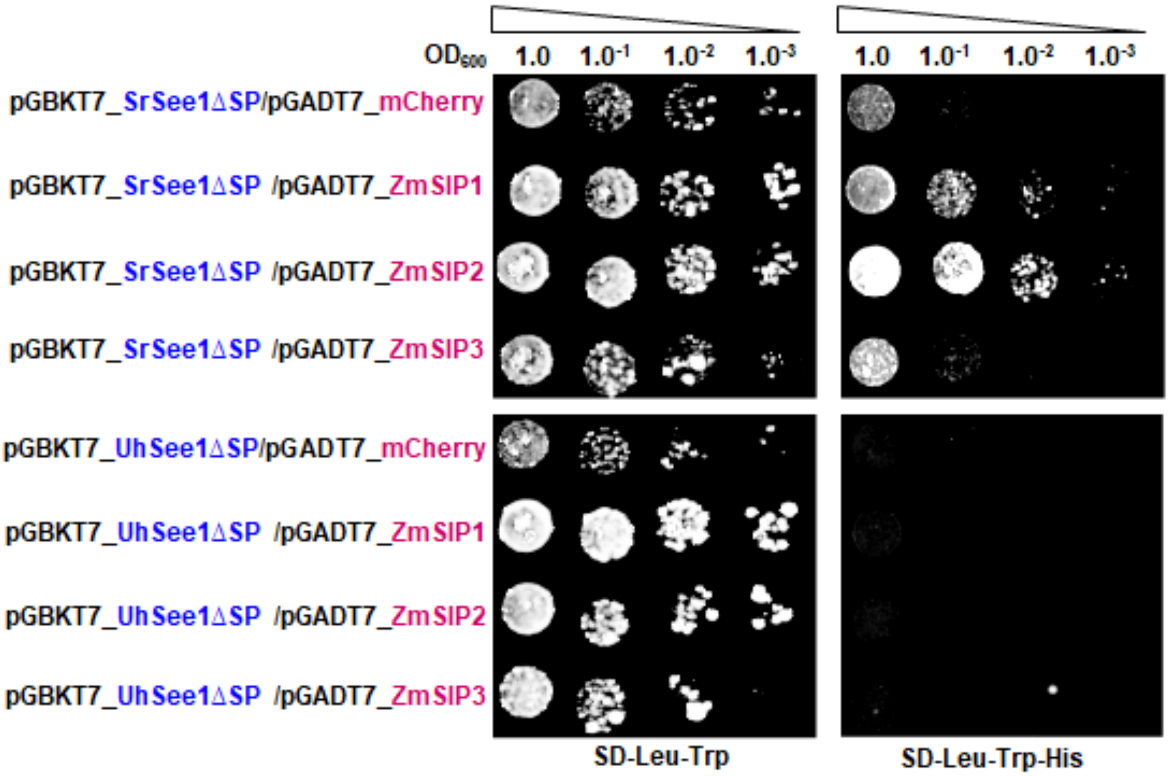

Fig S6.: Y2H assays involving SrSee1 and UhSee1 and the ZmSIPs. Yeast strain AH109 was co-transformed with constructs of ZmSIPs (ZmSIP1, ZmSIP2, ZmSIP3) and mCherry, SrSee1 or UhSee1 lacking signal peptide as indicated, and a drop-out series of the double-transformants was grown in the presence (SD-Trp-Leu) and absence (SD-Trp-Leu-His) of histidine. Yeast growth in the absence of histidine suggests the interaction of proteins encoded at the plasmids transformed. Pictures of three independent experiments were taken 5 days after drop out.

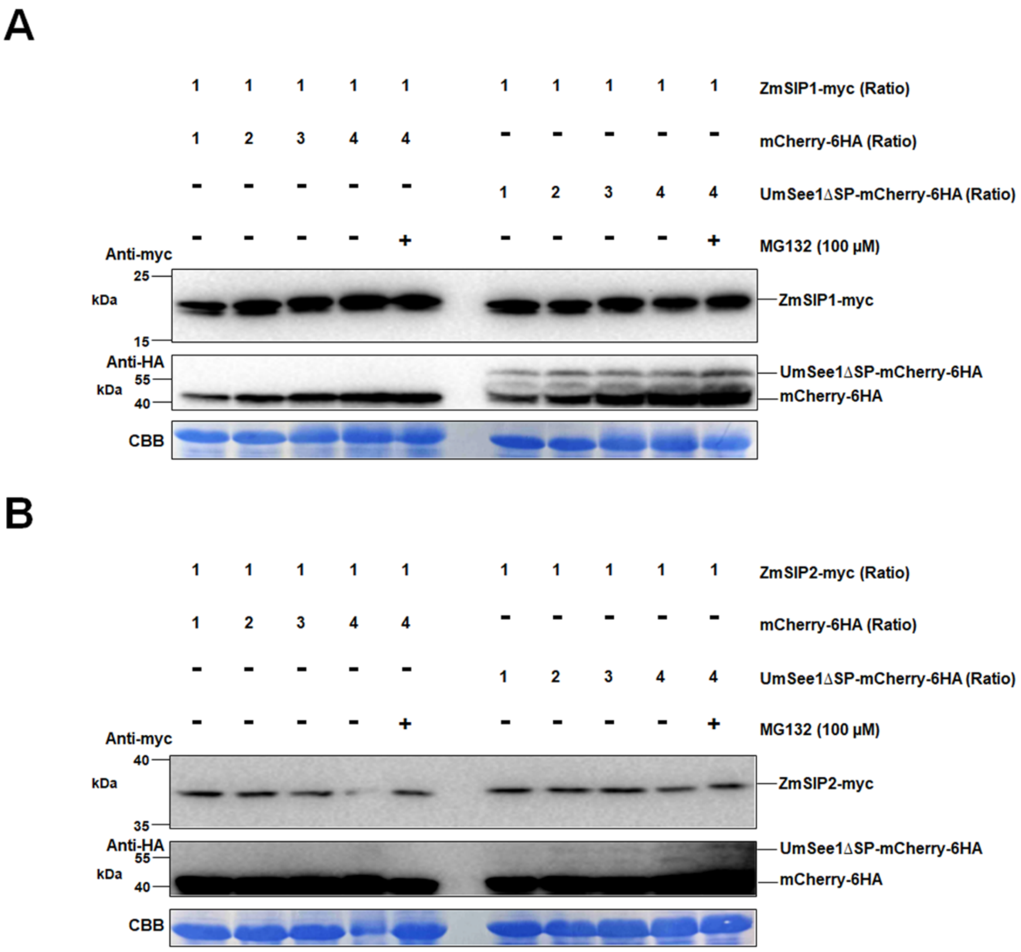

Fig S7.: In vitro protein degradation analysis of ZmSIP1 and ZmSIP2. (**A**) ZmSIP1-4myc, mCherry-6HA and UmSee1ΔSP-mCherry-6HA were separately expressed in *N. benthamiana* leaves. Total ZmSIP1-4myc protein lacking protease inhibitor were mixed in different proportions with mCherry-6HA or UmSee1ΔSP-mCherry-6HA protein extracts either in the presence of 100 μM MG132 or DMSO (control) and incubated at 28°C for 45 min before the reactions were stopped by boiling samples at 95°C in 2xSDS loading buffer for 10 min. Samples were analyzed by western blot using anti-myc and anti-HA as indicated. Coomassie brilliant blue (CBB) staining served as loading control. (**B**) ZmSIP2-4myc, mCherry-6HA and UmSee1ΔSP-mCherry-6HA were separately expressed in *N. benthamiana* leaves. Total ZmSIP2-4myc protein lacking protease inhibitor were mixed in different proportions with mCherry-6HA or UmSee1ΔSP-mCherry-6HA protein extracts either in the presence of 100 μM MG132 or DMSO (control) and incubated at 28°C for 45 min before the reactions were stopped by boiling samples at 95°C in 2xSDS loading buffer for 10 min. Samples were analyzed by western blot using anti-myc and anti-HA as indicated. Coomassie brilliant blue (CBB) staining served as loading control.
