# Supplementary material for: Combination of in vivo proximity labeling and co-immunoprecipitation identifies the host target network of a tumor-inducing effector in the fungal maize pathogen Ustilago maydis
Source: J Exp Bot. 2023 May 24;74(15):4736–50. doi: 10.1093/jxb/erad188 (PMC10433927; doi:10.1093/jxb/erad188)
Supplement: erad188_suppl_Supplementary_Tables_S1-S3 [file erad188_suppl_supplementary_tables_s1-s3.pdf]

Table S1: Primers used in this study.

Primer	Sequence (5'-3')	Purpose
TurboID-F	cgggcggccgctagctctagaATGAAAGACAATACTGT	ORF of TurboID
TurboID-R	ctgcagccgggcggctctagaTTAAGCGTAATCTGGAACAT	ORF of TurboID
mCherry-F	cgcagcaccgcggatcccATGGTGAGCAAGGGCGA	ORF of mCherry
mCherry-R	tagagctagcggccgcccgCTTGTACAGCTCGTCCA	ORF of mCherry
SP_{umsee1}-F	cgaccaaacgcagcaccgcggATGCTCTTCACCACCTT	Signal peptide of UmSee1
SP_{umsee1}-R	cctcgcccttgctcaccatggGAGCAGACACGTGGACAA	Signal peptide of UmSee1
UmSee1-R	tagagctagcggccgcccCCGTCGTCGGCCCAAATT	ORF of UmSee1
ZmSGT1-F	AGGCCTggatccTCGCGAatgGCCGCGTCGGATCTG	ORF of ZmSGT1
ZmSGT1-R	ATGGTGATGATGACTAGTAATTTCCCACTTCTTGAG	ORF of ZmSGT1
UmSee1-F1	AGGCCTggatccTCGCGAatgCATCCTCTACAATCG	ORF of UmSee1
UmSee1-R2	ATGGTGATGATGACTAGTCGTCGTCGGCCCAAATTT	ORF of UmSee1
SrSee1-F	AGGCCTggatccTCGCGAATGCGACCCACCAACACT	ORF of SrSee1
SrSee1-R	ATGGTGATGATGACTAGTCGTGTACGAATCGCCCAC	ORF of SrSee1
UhSee1-F	AGGCCTggatccTCGCGAATGCTCCCACCACGCCAC	ORF of UhSee1
UhSee1-R	ATGGTGATGATGACTAGTACCCCCCTCTCCGCCTCT	ORF of UhSee1
See1-NcoI-F	CATCCATGGCCATGATGCATCCGCTGCAG	ORF of UmSee1
See1-XhoI-R	CATCTCGAGTTAGGTTGTAGGACCAAAC	ORF of UmSee1
ZmSIP1-F	AGGCCTggatccTCGCGAATGGCCAACAGCAACCTC	ORF of ZmSIP1
ZmSIP1-R	ATGGTGATGATGACTAGTTGCACCGCTCGCATACAG	ORF of ZmSIP1
ZmSIP2-F	AGGCCTggatccTCGCGAATGAGCAGCATAGGCACA	ORF of ZmSIP2
ZmSIP2-R	ATGGTGATGATGACTAGTGTCAGCATCCATCTCCTC	ORF of ZmSIP2
ZmSIP3-F	AGGCCTggatccTCGCGAATGGCGGGAAGGTATCAC	ORF of ZmSIP3
ZmSIP3-R	ATGGTGATGATGACTAGTGTAACGGATAATATCACT	ORF of ZmSIP3

Table S2: *Ustilago maydis* strains used in this study.

Strain	Reference
SG200	Kamper *et al.*, 2006
ΔSee1	Redkar *et al.*, 2015
mCherry-TurboID-HA/SG200	This work
UmSee1-TurboID-HA/ΔSee1	This work

Reference:

Kamper J, Kahmann R, Bolker M, Ma LJ, Brefort T, Saville BJ, Banuett F, Kronstad JW, Gold SE, Muller O, Perlin MH, Wosten HA, de Vries R, Ruiz-Herrera J, Reynaga-Pena CG, Snetselaar K, McCann M, Perez-Martin J, Feldbrugge M, Basse CW, Steinberg G, Ibeas JI, Holloman W, Guzman P, Farman M, Stajich JE, Sentandreu R, Gonzalez-Prieto JM, Kennell JC, Molina L, Schirawski J, Mendoza-Mendoza A, Greilinger D, Munch K, Rossel N, Scherer M, Vranes M, Ladendorf O, Vincon V, Fuchs U, Sandrock B, Meng S, Ho EC, Cahill MJ, Boyce KJ, Klose J, Klosterman SJ, Deelstra HJ, Ortiz-Castellanos L, Li W, Sanchez-Alonso P, Schreier PH, Hauser-Hahn I, Vaupel M, Koopmann E, Friedrich G, Voss H, Schluter T, Margolis J, Platt D, Swimmer C, Gnirke A, Chen F, Vysotskaia V, Mannhaupt G, Guldener U, Munsterkotter M, Haase D, Oesterheld M, Mewes HW, Mauceli EW, DeCaprio D, Wade CM, Butler J, Young S, Jaffe DB, Calvo S, Nusbaum C, Galagan J, Birren BW. 2006. Insights from the genome of the biotrophic fungal plant pathogen *Ustilago maydis*. Nature 444, 97-101.

Redkar A, Hoser R, Schilling L, Zechmann B, Krzymowska M, Walbot V, Doehlemann G. 2015. A secreted effector protein of *Ustilago maydis* guides maize leaf cells to form tumors. Plant Cell 27, 1332-1351.

Table S3 Quantification of infection symptoms on maize seedlings at 12 dpi.

	Disease Index	0	1	3	5	7	9	11			
Replicate	Strain	No symptoms	Chlorosis	Swelling	Small tumors	Normal tumors	Heavy tumors	Dead plants	Total plants	Total disease index	Average disease index
1	SG200	0	0	6	14	32	8	1	61	**395**	6,475409836
1	mCherry-TurboID-3HA/SG200	0	0	4	8	26	10	0	48	**324**	6,75
1	ΔSee1	0	1	2	35	22	2	2	64	**376**	5,875
1	UmSee1-TurboID-3HA/ΔSee1	0	1	1	9	23	15	1	50	**356**	7,12
2	SG200	1	2	0	10	28	9	0	50	**329**	6,58
2	mCherry-TurboID-3HA/SG200	0	5	0	5	29	6	4	49	**331**	6,755102041
2	ΔSee1	0	0	5	27	14	4	1	51	**295**	5,784313725
2	UmSee1-TurboID-3HA/ΔSee1	1	1	1	13	19	14	1	50	**339**	6,78
3	SG200	0	3	0	10	12	8	4	37	**253**	6,837837838
3	mCherry-TurboID-3HA/SG200	0	2	1	10	23	6	5	47	**325**	6,914893617
3	ΔSee1	0	6	1	21	8	3	4	43	**241**	5,604651163
3	UmSee1-TurboID-3HA/ΔSee1	0	1	0	11	15	10	2	39	**273**	7

Strain	No symptoms	Chlorosis	Swelling	Small tumors	Normal tumors	Heavy tumors	Dead plants	Total plants
**SG200**	0,68%	3,38%	4,05%	22,97%	48,65%	16,89%	3,38%	**148**
**mCherry-TurboID-3HA/SG200**	0,00%	4,86%	3,47%	15,97%	54,17%	15,28%	6,25%	**144**
**ΔSee1**	0,00%	4,43%	5,06%	52,53%	27,85%	5,70%	4,43%	**158**
**UmSee1-TurboID-3HA/ΔSee1**	0,72%	2,16%	1,44%	23,74%	41,01%	28,06%	2,88%	**139**

Strain	disease index 1	disease index 2	disease index 3	Mean	Std
**SG200**	6,47541	6,58	6,837838	**6,631083**	**0,186536**
**mCherry-TurboID-3HA/SG200**	6,75	6,755102	6,914894	**6,806665**	**0,093763**
**ΔSee1**	5,875	5,784314	5,604651	**5,754655**	**0,137593**
**UmSee1-TurboID-3HA/ΔSee1**	7,12	6,78	7	**6,966667**	**0,172434**

Dunnett's multiple comparisons test	Mean Diff.	95.00% CI of diff.	Below threshold?	Summary	Adjusted P Value
SG200 vs. mCherry-TurboID-3HA/SG200	-0,1756	-0.5327 to 0.1815	No	ns	0,4036
SG200 vs. ΔSee1	0,8764	0.5193 to 1.234	Yes	***	0,0003
SG200 vs. UmSee1-TurboID-3HA/ΔSee1	-0,3356	-0.6927 to 0.02150	No	ns	0,0647
